# OpenCap-based five-times sit-to-stand analysis in older Asian women with and without sarcopenia

**DOI:** 10.3389/fragi.2026.1960201

**Published:** 2026-10-05

**Authors:** Jialiang Xu, Hejin Cai, Yan Wang, Zhengsheng Hu, Ming Zhang, Qitao Tan

**Affiliations:** 1 Department of Biomedical Engineering, Faculty of Engineering, The Hong Kong Polytechnic University, Hong Kong, Hong Kong SAR, China; 2 Research Institute for Sports Science and Technology, The Hong Kong Polytechnic University, Hong Kong, Hong Kong SAR, China

**Keywords:** biomechanics, five-times sit-to-stand, kinematic analysis, OpenCap, sarcopenia

## Abstract

**Introduction:**

Sarcopenia‐related lower‐limb functional impairment is commonly assessed using five‐times sit‐to‐stand (5STS) completion time, which provides limited information about how performance differs within the task. This study examined two questions: the concurrent agreement of OpenCap‐derived lower‐limb kinematics with Vicon during repeated 5STS, and whether OpenCap‐derived kinematic and temporal measures distinguished healthy and sarcopenic older Asian women.

**Methods:**

Twenty‐four participants were classified as healthy (n = 12) or sarcopenic (n = 12) according to AWGS 2025 criteria and completed concurrent OpenCap and Vicon recordings. Agreement was assessed from participant‐level mean waveforms using RMSE and MAE for angular error magnitude, ICC(A,1) for absolute agreement, R^2^ for waveform similarity, and phase‐dependent bias.

**Results:**

Knee flexion/extension showed the lowest absolute error and high agreement (RMSE = 1.84°, ICC(A,1) = 0.998). Hip flexion/extension also showed high agreement, although absolute error was larger (RMSE = 5.42°, ICC(A,1) = 0.970). Ankle agreement was more variable. Statistical parametric mapping identified localized reductions in hip and knee flexion in the sarcopenic group, while angular velocity waveforms did not differ significantly. Sarcopenic participants also showed longer total, ascent, and descent durations and greater repetition-related slowing, while repetition-duration variability did not differ significantly.

**Discussion:**

Within this protocol, OpenCap showed strong participant‐level agreement for hip and knee kinematics and provided phase‐ and repetition‐specific descriptors of 5STS performance. The independent validity and incremental clinical value of the derived temporal measures require further evaluation.

## 1 Introduction

Sarcopenia is an age-associated decline in skeletal muscle mass and function that contributes to frailty, falls, mobility limitation, and disability in older adults (Yeung et al., 2019; Beaudart et al., 2017; Chen et al., 2025). It is therefore a major public health concern (Bruyère et al., 2019), with reported prevalence ranging from 10% to 40% in older populations (Mayhew et al., 2019; Petermann-Rocha et al., 2022). Prevalence estimates and diagnostic thresholds vary across populations and by sex, and the Asian Working Group for Sarcopenia (AWGS) applies sex-specific criteria for muscle mass and strength in Asian adults (Chen et al., 2025). Early identification enables timely nutritional and rehabilitation interventions. However, clinical assessment remains challenging. The SARC-F questionnaire is simple but subjective, and it can miss early or mild impairment (Malmstrom and Morley, 2013). Confirmatory assessment by dual-energy X-ray absorptiometry or bioelectrical impedance analysis (BIA) requires dedicated equipment and trained personnel. These requirements limit access in community and remote healthcare settings (Han et al., 2018).

Performance-based tests provide accessible, low-cost, and interpretable measures of function. Major sarcopenia guidelines, including the Asian Working Group for Sarcopenia (AWGS) and the European Working Group on Sarcopenia, recommend the five-times sit-to-stand (5STS), also referred to as the five-time chair stand test, as a practical measure of lower-limb physical performance (Chen et al., 2025; Cruz-Jentoft et al., 2019). Routine assessment usually records only total completion time using a stopwatch. However, the 5STS is a repeated motor task in which each repetition requires forward transfer of body mass, rising from the chair, stabilization in upright stance, controlled lowering, and reseating. These sequential components place different demands on lower-limb force generation, postural control, balance, and neuromuscular coordination (Schenkman et al., 1990; Sadeh et al., 2023; Promsri and Federolf, 2026). They also reflect transfers that are routinely performed during daily activities. Total completion time summarizes overall task performance but does not identify which component of the movement contributes to impaired performance.

Sarcopenia-related functional impairment can appear as altered joint excursions, phase-specific slowing, progressive changes in pace across repetitions, and compensatory movement strategies (Coelho-Júnior et al., 2024; Cai et al., 2025). Individuals with similar total 5STS completion times can perform the task through different movement profiles, such as prolonged rising, cautious descent, progressive slowing, or altered joint excursion. Joint-, phase-, and repetition-specific analysis can characterize these differences and identify where functional limitations occur within the task. Laboratory-based three-dimensional motion capture remains the reference standard for this analysis. Its dependence on reflective markers, specialized equipment, trained operators, and dedicated space restricts use in community and large-scale screening settings (Philipp et al., 2024).

Recent advances in markerless motion capture and computer vision offer a scalable route for biomechanical assessment by extracting video-based kinematics, minimizing the need for complicated laboratory setups and instrumentation (Wang et al., 2024; Lam et al., 2023; Wade et al., 2022). Building on these capabilities, the platform OpenCap combines synchronized smartphone videos with musculoskeletal modeling to estimate three-dimensional human movement (Uhlrich et al., 2023). This portable design supports clinical and community assessment, but validation remains specific to each task, joint, and population (Uhlrich et al., 2023; Turner et al., 2024). OpenCap has already been applied to gait assessment in knee osteoarthritis and mixed neurological cohorts including stroke, Parkinson’s disease, cerebral palsy, Guillain–Barré syndrome, and cerebellar ataxia, as well as to standing-balance assessment in children with cerebral palsy (Wang et al., 2025; Min et al., 2024; Yan et al., 2025). Previous studies have evaluated OpenCap during gait, sport, and other functional movements (Turner et al., 2024; Svetek et al., 2025). Video-based approaches have also been explored for sarcopenia assessment using sit-to-stand performance (Ruiz-Cárdenas et al., 2023). These studies establish the broader feasibility of video-based movement assessment, while leaving two questions unresolved for 5STS in sarcopenia: whether OpenCap reproduces joint-level kinematics relative to laboratory motion capture and whether its derived measures can characterize where functional differences occur within the task. The contribution of the present study is task- and population-specific validation of an established markerless motion capture platform together with joint-, phase-, and repetition-level characterization of 5STS performance. Because AWGS 2025 applies sex-specific thresholds for muscle mass and strength, the present validation cohort was restricted to older Asian women to maintain a homogeneous sample within this sex-specific classification framework (Chen et al., 2025). The resulting scope of inference is therefore limited to older Asian women.

Therefore, this study addressed two complementary measurement-property questions. First, concurrent validity was evaluated by examining the agreement between OpenCap-derived lower-limb flexion–extension kinematics and Vicon during repeated 5STS. Second, known-groups validity was examined by determining whether OpenCap-derived joint waveforms, phase durations, and repetition-related temporal measures differed between healthy and sarcopenic older women independently classified according to the AWGS 2025 criteria (Figure 1). We hypothesized that OpenCap would demonstrate acceptable agreement with Vicon and that the sarcopenic group would exhibit altered lower-limb kinematic waveforms, prolonged phase durations, and greater repetition-related slowing.

**FIGURE 1:**
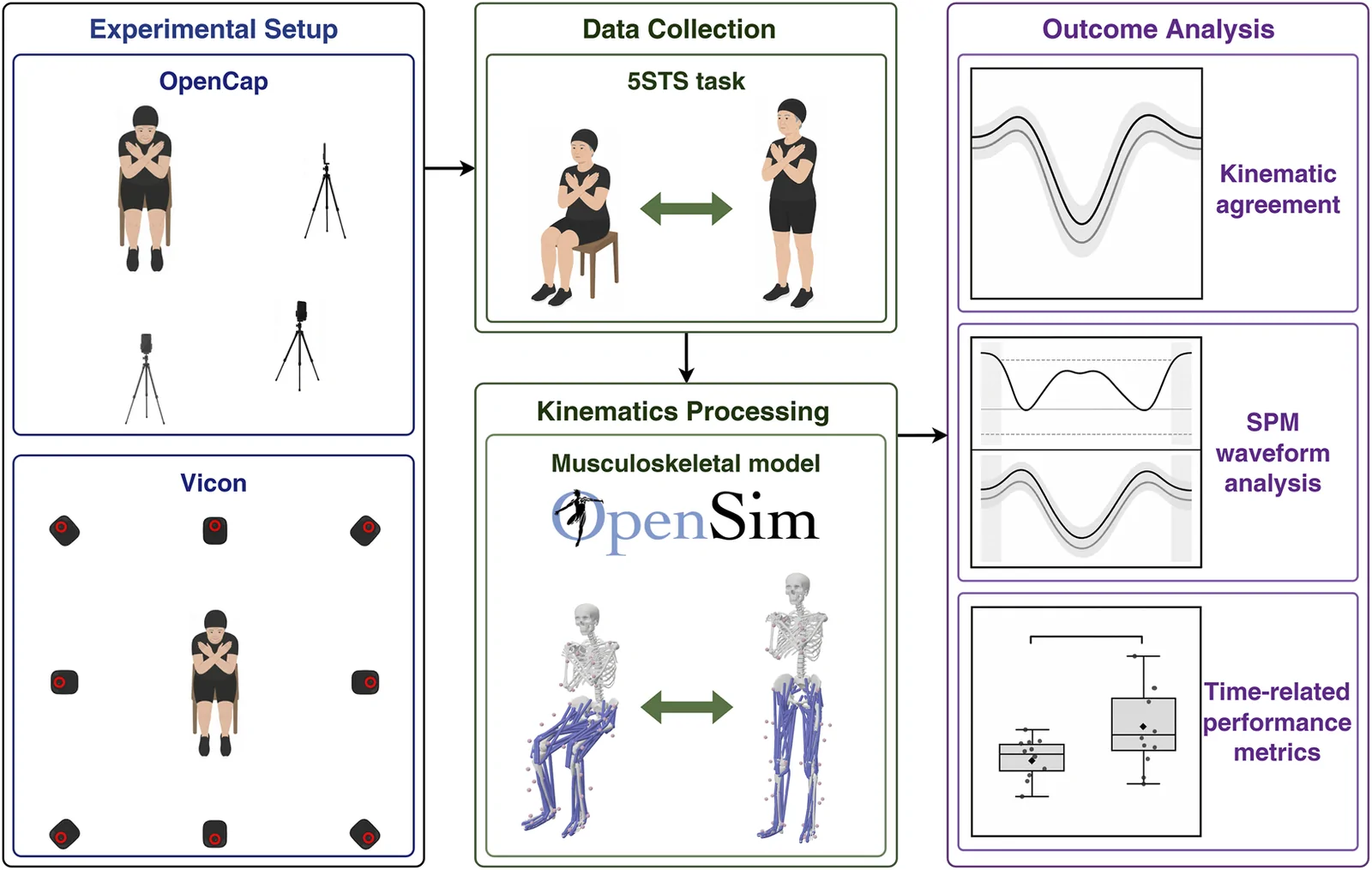
Overview of the study workflow. The analysis included lower-limb kinematic processing, OpenCap–Vicon agreement assessment, statistical parametric mapping of joint waveforms, and comparison of time-related 5STS performance metrics. Abbreviations: 5STS, five-times sit-to-stand. SPM, statistical parametric mapping.

## 2 Materials and methods

### 2.1 Participants

Twenty-four older adults were recruited through posters displayed on the university campus. Data were collected from mid-November through December 2025. All participants were Asian women aged 65 years or older who could walk independently and perform 5STS without assistance. Participants were excluded if they had conditions affecting functional performance (stroke, Parkinson’s disease, lower limb deformities, osteoarthritis, severe back pain, or recent surgery) or comorbidities representing risk factors or confounders (uncontrolled cardiovascular or renal disease, visible edema, implanted cardiac pacemaker, cognitive impairment, or severe visual/hearing impairment). A screening questionnaire collected information on habitual physical activity, fall history, medication use, lower-limb dominance, pain, and visual or balance problems to identify potential factors affecting 5STS performance. No formal frailty classification or standardized physical-activity assessment was performed. These screening variables were not used for AWGS 2025 group classification or included as covariates in the inferential analyses.

Participants were classified into sarcopenic and healthy groups according to the AWGS 2025 criteria (Chen et al., 2025). Appendicular skeletal muscle mass (ASM) was assessed using BIA (InBody 970, InBody Co. Ltd., Seoul, South Korea). Handgrip strength was measured using a handheld dynamometer (CAMRY EH101, Zhongshan Camry Electronic Co. Ltd., Zhongshan, China). The maximum value from three trials with each hand was recorded. BIA is an accepted method for estimating muscle mass within the AWGS framework, and the CAMRY EH101 has demonstrated good reliability and concurrent validity for handgrip-strength assessment in older adults (Chen et al., 2025; Huang et al., 2022). Calf circumference was measured while participants stood naturally using a non-elastic measuring tape. The maximum circumference of each calf was measured separately, and the larger of the left and right values was retained as the participant-level calf circumference measure (Piodena-Aportadera et al., 2022). Skeletal muscle mass index (SMI) was calculated as ASM divided by height squared. The sarcopenic classification required both low muscle strength and low muscle mass, defined as handgrip strength <18.0 kg and the height-adjusted BIA criterion of SMI <5.7 kg/ $m^{2}$ . The healthy classification required handgrip strength ≥18.0 kg and SMI ≥5.7 kg/ $m^{2}$ . These sex-specific cut-offs were specified by the AWGS 2025 consensus. Only participants meeting both criteria for one of the two predefined groups were included in the analysis. Participants with discordant muscle-mass and handgrip-strength profiles were excluded. Table 1 summarizes participant characteristics.

**TABLE 1:** Baseline characteristics of study participants categorized by AWGS 2025 criteria.

Variable	Healthy (n=12)	Sarcopenic (n=12)	Effect size	p -value
Age (years)	69.08±3.58	70.17±2.79	0.34	$0.418^{a}$
Height (m)	1.56±0.04	1.53±0.05	0.53	$0.205^{a}$
BMI (kg/ $m^{2}$ )	18.61±1.58	18.36±1.93	0.15	$0.727^{a}$
ASM (kg)	14.05±0.88	12.03±1.01	2.14	**<** 0.001 **^a^**
SMI (kg/ $m^{2}$ )	5.80±0.14	5.12±0.19	4.05	**<** 0.001 **^a^**
Handgrip strength (kg)	21.21±2.12	14.67±2.68	2.71	**<** 0.001 **^a^**
Weight (kg)	44.90 [40.85, 50.35]	40.70 [39.60, 45.60]	0.34	$0.165^{b}$
Calf circumference (cm)	33.27±1.87	30.77±0.95	1.68	**<** 0.001 **^a^**

Normally distributed variables are presented as mean ± standard deviation (SD) and were compared using Welch’s two-sample t -test, with effect sizes reported as Cohen’s d . Non-normally distributed variables are presented as median [interquartile range] and were compared using the Mann–Whitney U test, with effect sizes reported as rank-biserial correlations. Normality was assessed using the Shapiro–Wilk test. Superscript a indicates Welch’s two-sample t -test and superscript b indicates the Mann–Whitney U test. Bold values indicate statistical significance (p<0.05) .

*Abbreviations:* BMI, body mass index. ASM, appendicular skeletal muscle mass. SMI, skeletal muscle mass index. AWGS, Asian Working Group for Sarcopenia.

This study was approved by the Human Subjects Ethics Subcommittee of The Hong Kong Polytechnic University (Ref. No. HSEARS20240625002), and written informed consent was obtained from all participants prior to data collection.

### 2.2 Test protocol

All experiments were conducted in a controlled laboratory environment. All participants wore the same standardized attire during data collection, consisting of a close-fitting black T-shirt, close-fitting black shorts, and standardized athletic shoes of the same model. The same clothing and footwear protocol was used for both groups and across all trials. This attire was selected to permit consistent placement of the Vicon markers while maintaining clearly visible body contours for OpenCap pose estimation.

Participants were seated on a standardized armless chair with a seat height of 43 cm, consistent with commonly used sit-to-stand protocols (Kuo, 2013). The arms were crossed over the chest, and participants self-selected a comfortable foot position approximately shoulder-width apart. After a verbal start cue, participants were instructed to stand fully and sit down five times as quickly as possible. Before the recorded trials, participants received standardized task instructions and completed one familiarization trial of the 5STS task, which was excluded from all analyses. Participants were allowed a self-selected seated rest interval between trials and proceeded only when they reported being ready. Rest duration was not formally recorded.

Twenty-four participants completed four trials each, yielding 96 recorded trials. Eight trials were excluded because OpenCap synchronization problems resulted in failed pose tracking and unusable kinematic outputs. The remaining 88 trials were included in the analysis.

### 2.3 Motion capture systems

Marker-based kinematic data were acquired at 250 Hz using an eight-camera optical motion capture system (Vicon Motion System, Oxford Metrics Ltd., Oxford, UK). Thirty-nine reflective markers were attached to the relevant anatomical locations on the participants’ exposed skin, standardized clothing and footwear, according to the Plug-in Gait full-body marker set (Vicon Motion Systems Ltd, 2024). Vicon served as the laboratory reference for OpenCap kinematics validation.

Three tripod-mounted iPhone 14 devices (Apple Inc., Cupertino, CA, United States of America) recorded videos at 720p and 60 fps, and the three video streams were automatically time-synchronized through the standard OpenCap multi-camera processing workflow (Uhlrich et al., 2023). The OpenCap pipeline processed the videos using the HRNet pose estimation algorithm (Cheng et al., 2020; Sun et al., 2019; Uhlrich et al., 2023). Cameras were calibrated with a 4×5 checkerboard that had 35 mm squares as officially recommended by OpenCap. Each device was positioned 3 m from the participant and 1.5 m above the floor. The cameras provided frontal (0°) , left-anterior oblique (approximately 45° ), and left-lateral (90°) views relative to the participant’s facing direction during the 5STS task. OpenCap and Vicon recordings were initiated near-simultaneously by two operators following a shared verbal countdown and then recorded concurrently.

### 2.4 Kinematic processing

OpenCap videos were processed using the standard OpenCap pipeline to generate subject-specific musculoskeletal models and estimate lower-limb kinematics. Marker-based Vicon data were processed in OpenSim 4.5 (Seth et al., 2018) using a corresponding musculoskeletal model to obtain reference joint kinematics. To reduce differences caused by model scaling and anatomical frame definitions, OpenCap model geometry (LaiUhlrich2022 geometry) was used as the common anatomical reference for both markerless and marker-based inverse kinematics analyses (Lai et al., 2017; Rajagopal et al., 2016; Uhlrich et al., 2023).

Hip flexion/extension, knee flexion/extension, and ankle dorsiflexion/plantarflexion angles were extracted from both systems because they represent the primary lower-limb joint excursions during sit–stand–sit movements (Lin and Lee, 2022; Anan et al., 2017). Custom Python scripts (3.12.0) and the OpenSim Inverse Kinematics Tool processed all kinematic data (Seth et al., 2018). A fourth-order, zero-lag Butterworth low-pass filter with a 6 Hz cutoff was applied uniformly to the OpenCap and Vicon joint-angle trajectories to attenuate high-frequency noise while preserving the principal movement dynamics and avoiding phase shifts during temporal alignment and phase-specific waveform analysis (Sher et al., 2026; Moreira et al., 2025). Vicon data were downsampled from 250 Hz to 60 Hz to match the OpenCap sampling frequency.

Cross-system temporal alignment was then performed separately for each complete 5STS trial, before repetition segmentation, time normalization, and participant-level waveform averaging, using the full cross-correlation between the OpenCap and Vicon left knee flexion/extension waveforms (Buchanan et al., 2026). For calculation of the cross-correlation, both signals were mean-centered and standardized. The lag corresponding to the maximum cross-correlation was applied uniformly to all joint-angle signals from the same trial, and non-overlapping samples were subsequently trimmed. The left knee waveform was selected because repeated sit–stand–sit movements produced a large-amplitude, clearly periodic sagittal-plane signal with relatively low noise, enabling consistent identification of temporal offsets across trials. This procedure corrected only the temporal lag between the two systems and did not rescale, warp, or otherwise modify the original joint-angle amplitudes.

### 2.5 Statistical analysis

One 5STS trial consisted of five consecutive sit–stand–sit repetitions. Each repetition began in the seated position, passed through upright standing, and ended at the subsequent kinematically defined seated configuration. Left and right joint-angle trajectories were averaged pointwise to generate one bilateral sagittal-plane waveform per joint for both OpenCap and Vicon. These waveforms represent task-level summaries of the standardized bilateral 5STS movement and should not be interpreted as evidence of limb symmetry or as side-specific measures. Each valid repetition was normalized to 0%–100% of the sit–stand–sit cycle. After normalization, all valid repetitions from all trials were averaged within each participant to form the participant-level mean waveform used in the primary validation analysis. The participant was treated as the independent analysis unit. Trials, repetitions, and waveform time points were not treated as independent observations. This averaging reduces within-participant random variability, so the reported agreement estimates characterize participant-level mean waveforms and should not be interpreted as single-trial or single-repetition measurement accuracy.

#### 2.5.1 Baseline characteristics

Baseline characteristics were summarized separately for the healthy and sarcopenic groups. The Shapiro–Wilk test assessed normality. Normally distributed variables were reported as mean ± standard deviation (SD) and compared using Welch’s two-sample t -test. Cohen’s d quantified effect size. Non-normally distributed variables were reported as median [interquartile range] and compared using the Mann–Whitney U test. Rank-biserial correlation quantified effect size. Statistical significance was set at p<0.05 .

#### 2.5.2 Validation metrics

Agreement between OpenCap and Vicon was evaluated using root mean square error (RMSE), mean absolute error (MAE), and ICC(A,1). The coefficient of determination $\left(R^{2}\right)$ was included as a supplementary measure of waveform similarity. RMSE and MAE quantified the magnitude of angular error in degrees, ICC(A,1) quantified absolute agreement, and $R^{2}$ quantified similarity in waveform variation.

ICC(A,1) was calculated as a two-way random-effects, absolute-agreement, single-measure intraclass correlation coefficient, corresponding to ICC(2,1) in the Shrout–Fleiss notation (McGraw and Wong, 1996; Koo and Li, 2016). For each participant and joint, normalized waveform phase points were treated as targets, and OpenCap and Vicon were treated as the two measurement systems. A single-measure ICC was selected because each system provided one measurement waveform rather than an average of multiple measurements. Absolute agreement was selected because systematic differences in joint-angle magnitude were considered measurement disagreement and should reduce the validity estimate. ICC(A,1) values were classified as poor (<0.50) , moderate (0.50–0.74), good (0.75–0.90), or excellent (>0.90) (Koo and Li, 2016).

All primary agreement metrics were calculated for each participant from the participant-level mean waveform before group-level summarization and are reported as mean estimates with 95% confidence intervals. Confidence intervals were obtained using bias-corrected and accelerated bootstrap resampling with 10,000 resamples and participants as the resampling unit. Mean bias was defined as OpenCap minus Vicon, and limits of agreement were calculated as the mean participant-level bias ±1.96 standard deviations. Bootstrap 95% confidence intervals were calculated for the mean bias and limits of agreement. Phase-dependent bias curves with pointwise 95% confidence intervals were also calculated.

#### 2.5.3 Statistical parametric mapping

Statistical parametric mapping (SPM) was used to examine group differences in OpenCap kinematic waveforms across the normalized sit–stand–sit cycle. Angular velocity was derived from the filtered joint-angle trajectories using first-order forward differences between adjacent samples and their corresponding time intervals. Absolute angular velocity was retained at each time point to characterize movement-velocity magnitude independent of flexion/extension direction. The resulting angular-velocity waveforms were time-normalized to 0%–100% of the movement cycle for waveform analysis.

Independent two-sample SPM{ t } tests compared the healthy and sarcopenic groups using the open-source spm1d Python package (0.4.53) (Pataky, 2012). Analyses included hip and knee flexion angles and the absolute magnitudes of the corresponding angular velocities. All tests were two-tailed at α=0.05 . Levene’s test confirmed homogeneity of variance for the analyzed waveforms, so the equal-variance formulation was used. Random field theory (RFT) estimated a critical threshold across the 0%–100% movement trajectory and controlled the family-wise error rate. A trajectory segment that exceeded this threshold formed a statistically significant suprathreshold cluster. Linear interpolation estimated cluster endpoints as percentages of the normalized cycle. RFT-based inference controlled the family-wise error rate across the normalized trajectory separately for each biomechanical variable.

#### 2.5.4 Time-related 5STS performance metrics

OpenCap data were used to derive total 5STS completion time, repetition-duration coefficient of variation (CV), and mean ascent and descent durations for each participant. Total completion time was measured from the verbal start cue until the participant was fully seated after the fifth stand, following conventional clinical timing.

Individual sit–stand–sit cycles were segmented separately using a custom semi-automated algorithm applied to the filtered bilateral mean knee-flexion trajectory. Successive knee-flexion maxima defined the seated configurations, and the intervening minimum defined upright standing. Ascent was defined from the first seated maximum to the intervening minimum, and descent from that minimum to the subsequent seated maximum. Two trained assessors jointly reviewed all detected events, and questionable boundaries were resolved by consensus. The number of individual manual corrections was not prospectively logged. Independent inter-rater agreement was not calculated because event review was performed as a joint consensus quality-control procedure.

To summarize changes in repetition duration across the five repetitions, a repetition-duration slope was first calculated separately for each valid trial. Within each trial, a linear regression model used repetition number (1–5) as the independent variable and the corresponding five repetition durations as the dependent variable. The resulting trial-specific slopes, expressed in seconds per repetition, were then averaged across all valid trials within each participant to obtain one participant-level slope for the between-group analysis. A larger positive value indicated a greater increase in repetition duration across successive repetitions.

The Shapiro–Wilk test assessed normality for each metric, and Levene’s test assessed homogeneity of variance. Normally distributed data were compared using an independent-samples t -test. Welch’s correction was applied when variances were unequal. The Mann–Whitney U test was used when data were non-normal. Hedges’ g , together with its 95% confidence interval, quantified the standardized between-group effect size and accounted for the small sample.

## 3 Results

### 3.1 Participant characteristics

The analysis included 12 healthy and 12 sarcopenic participants. Age, height, BMI, and body weight did not differ significantly between groups (all p>0.05 ). The sarcopenic group had lower ASM, SMI, handgrip strength and calf circumference than the healthy group (all p<0.001 ), consistent with the group classification and body-composition characteristics (Table 1).

### 3.2 Agreement between OpenCap and Vicon kinematics during 5STS

Trial-specific temporal alignment was successfully obtained for all 88 included trials. The median absolute OpenCap–Vicon lag was 1.017 s [IQR: 0.613–1.387 s]. Because the two systems were started manually by separate operators without a shared hardware trigger, this offset is interpreted to primarily reflect differences in recording onset rather than a within-trial kinematic timing delay. OpenCap joint-angle waveforms followed the corresponding Vicon waveforms across the sit–stand–sit cycle in both groups (Figure 2A). Table 2 summarizes the agreement metrics. Knee flexion/extension showed the lowest absolute error (RMSE = 1.84° , MAE = 1.52° ) and high absolute agreement [ICC(A,1) = 0.998]. Hip flexion/extension also showed high agreement [ICC(A,1) = 0.970], although its absolute errors were larger (RMSE = 5.42° , MAE = 4.62° ). Ankle dorsiflexion/plantarflexion showed relatively small absolute errors (RMSE = 1.52° , MAE = 1.32° ) but more variable relative agreement [ICC(A,1) = 0.881]. $R^{2}$ values were 0.976, 0.998, and 0.846 for the hip, knee, and ankle, respectively.

**FIGURE 2:**
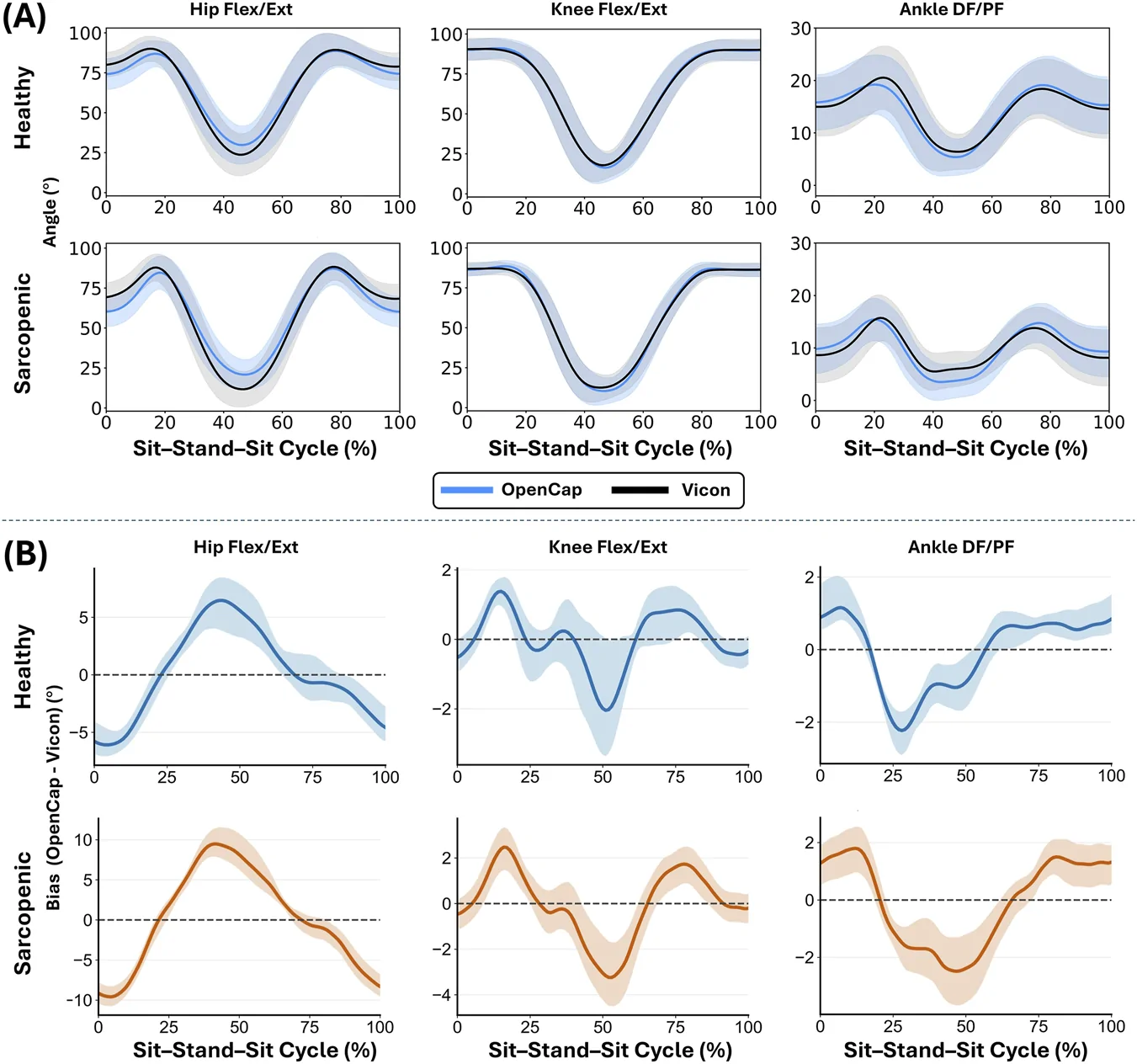
Agreement and phase-dependent bias between OpenCap and Vicon lower-limb joint angle waveforms during 5STS task. **(A)** Group-averaged hip flexion/extension, knee flexion/extension, and ankle dorsiflexion/plantarflexion waveforms for the healthy and sarcopenic groups. Blue lines represent OpenCap kinematics, black lines represent Vicon kinematics, and shaded regions indicate between-subject standard deviation (SD). **(B)** Phase-dependent bias, defined as OpenCap minus Vicon, for the same joints and groups. Blue and orange lines represent the participant-level mean bias in the healthy and sarcopenic groups, respectively. Shaded regions represent pointwise 95% confidence intervals calculated using bias-corrected and accelerated bootstrap resampling with 10,000 resamples and the participant as the resampling unit. Dashed horizontal lines indicate zero bias. All waveforms were time-normalized to 0%–100% of one sit–stand–sit cycle. Abbreviations: Flex, flexion. Ext, extension. DF, dorsiflexion. PF, plantarflexion.

**TABLE 2:** Comparison of OpenCap and Vicon kinematic agreement during the 5STS task.

Joint/Metric	Healthy (n=12)	Sarcopenic (n=12)	Overall (n=24)
Hip flexion/extension			
RMSE (°)	4.54 (3.77–5.32)	6.29 (5.28–7.45)	5.42 (4.72–6.25)
MAE (°)	3.83 (3.18–4.60)	5.42 (4.50–6.49)	4.62 (3.99–5.37)
ICC(A,1)	0.977 (0.965–0.983)	0.962 (0.942–0.974)	0.970 (0.958–0.977)
$R^{2}$	0.983 (0.972–0.989)	0.969 (0.961–0.976)	0.976 (0.969–0.982)
Knee flexion/extension			
RMSE (°)	1.56 (1.25–1.91)	2.13 (1.76–2.63)	1.84 (1.58–2.17)
MAE (°)	1.23 (1.00–1.50)	1.82 (1.50–2.26)	1.52 (1.30–1.81)
ICC(A,1)	0.998 (0.997–0.999)	0.997 (0.996–0.998)	0.998 (0.997–0.998)
$R^{2}$	0.998 (0.997–0.999)	0.998 (0.998–0.999)	0.998 (0.998–0.998)
Ankle dorsiflexion/plantarflexion			
RMSE (°)	1.23 (1.01–1.60)	1.81 (1.25–2.38)	1.52 (1.22–1.89)
MAE (°)	1.02 (0.83–1.41)	1.62 (1.10–2.14)	1.32 (1.03–1.67)
ICC(A,1)	0.953 (0.926–0.970)	0.809 (0.594–0.906)	0.881 (0.757–0.933)
$R^{2}$	0.930 (0.898–0.954)	0.762 (0.533–0.884)	0.846 (0.718–0.907)

Data are presented as participant-level mean estimates with 95% confidence intervals in parentheses. For each participant, all validation metrics were calculated from the participant-level mean waveform obtained by averaging all valid time-normalized repetitions. Confidence intervals were calculated using bias-corrected and accelerated bootstrap resampling with 10,000 resamples and the participant as the resampling unit.

*Abbreviations:* RMSE, root mean square error. MAE, mean absolute error. ICC, intraclass correlation coefficient. $R^{2}$ , coefficient of determination.

Cycle-averaged mean biases (OpenCap minus Vicon) were 0.19° for the hip, −0.02° for the knee, and −0.05° for the ankle. The corresponding limits of agreement were −0.03° to 0.41° , −0.09° to 0.05° , and −0.09° to 0.00° , respectively. Figure 2B shows the phase-dependent direction and uncertainty of OpenCap–Vicon differences across the movement cycle. Group-specific mean biases, limits of agreement with 95% confidence intervals, and complete participant-level ICC(A,1) distributions are provided in Supplementary Data S1.

### 3.3 Between-group differences in OpenCap kinematic waveforms

SPM detected significant between-group differences in the OpenCap hip and knee angle waveforms (Figure 3). For hip flexion/extension, the RFT critical threshold was $t^{*}=3.1051$ , and two suprathreshold clusters occurred at 0.0%–9.2% (extent: 9.18% of the normalized cycle; cluster-level p=0.0301 ) and 89.8%–100.0% (extent: 10.19%; cluster-level p=0.0267 ). The sarcopenic group had lower hip flexion angles in both regions. For knee flexion/extension, the critical threshold was $t^{*}=3.1330$ , with clusters at 36.5%–38.4% (extent: 1.92%; cluster-level p=0.0487 ) and 49.3%–50.2% (extent: 0.89%; cluster-level p=0.0497 ). These knee clusters were brief, and the sarcopenic group had lower knee flexion angles.

**FIGURE 3:**
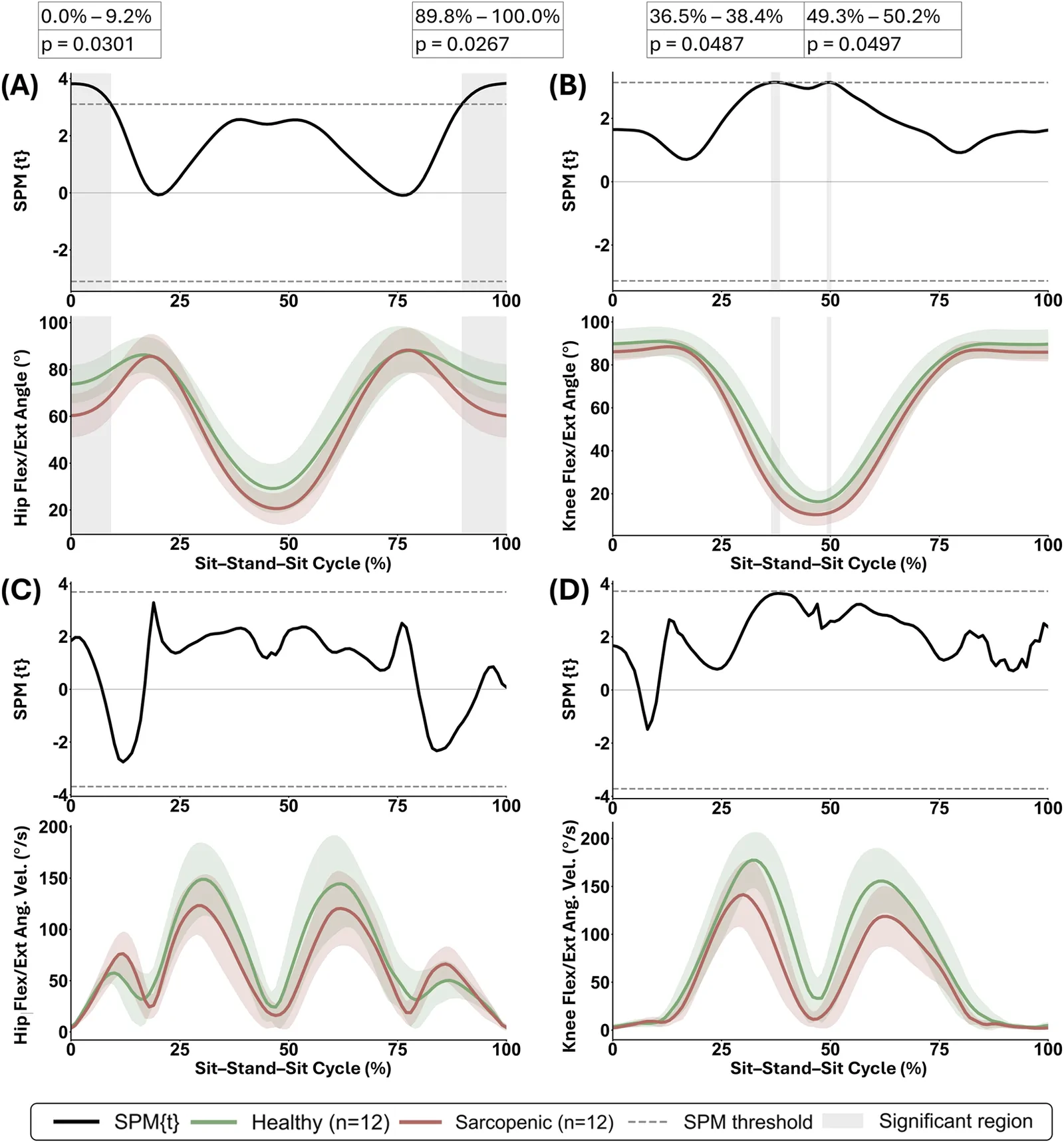
Statistical parametric mapping of OpenCap lower-limb kinematic waveforms during the 5STS task. Each panel contains an upper SPM{ t } trajectory and a lower group-mean waveform. Panels show **(A)** hip flexion/extension angle, **(B)** knee flexion/extension angle, **(C)** hip flexion/extension angular velocity, and **(D)** knee flexion/extension angular velocity. Dashed horizontal lines indicate the two-tailed RFT critical thresholds (α=0.05) , and grey-shaded regions denote statistically significant suprathreshold clusters. Colored lines represent group means, and shaded bands indicate between-subject SD. All waveforms were time-normalized to 0%–100% of one sit–stand–sit cycle. Angular velocity waveforms are presented as absolute magnitudes. Abbreviations: Ang. Vel., angular velocity. RFT, random field theory.

The absolute hip and knee angular velocity waveforms had critical thresholds of $t^{*}=3.6875$ and $t^{*}=3.7173$ , respectively, and neither contained a suprathreshold cluster. Group-mean peaks appeared lower in the sarcopenic group, but the visual differences did not constitute statistically significant waveform effects.

### 3.4 Between-group differences in time-related 5STS performance metrics

Table 3 and Figure 4 summarize the time-related performance metrics derived from the OpenCap data. The sarcopenic group had a longer total 5STS completion time than the healthy group ( 16.23±3.73 s vs. 10.65±1.46 s, p<0.001 , Hedges’ g=1.90 , 95% CI 0.94–2.87). The sarcopenic group also had a larger repetition-duration slope than the healthy group ( 0.09±0.08 s/rep vs. 0.04±0.04 s/rep, p=0.032 , Hedges’ g=0.93 , 95% CI 0.09–1.77).

**TABLE 3:** Comparison of OpenCap time-related 5STS performance metrics.

Metric	Healthy	Sarcopenic	Statistical test	Hedges’ g (95% CI)	p -value
5STS total time (s)	10.65±1.46	16.23±3.73	Student’s t -test	1.90 (0.94,2.87)	**<** 0.001
Repetition-duration slope (s/rep)	0.04±0.04	0.09±0.08	Welch’s t -test	0.93 (0.09,1.77)	**0.032**
Repetition-duration CV (%)	7.13±1.66	7.84±2.64	Student’s t -test	0.31 (−0.49,1.12)	0.438
Ascent phase duration (s)	0.98±0.16	1.48±0.33	Student’s t -test	1.89 (0.93,2.85)	**<** 0.001
Descent phase duration (s)	1.15±0.16	1.76±0.44	Student’s t -test	1.77 (0.83,2.71)	**<** 0.001

Data are presented as mean ± SD. The Shapiro–Wilk and Levene’s tests assessed normality and homogeneity of variance, respectively. All five metrics satisfied the normality criterion, so the Mann–Whitney U test was not required. Welch’s t -test was applied to repetition-duration slope because its variances were unequal, whereas Student’s t -test was applied to the remaining metrics. Hedges’ g and its 95% confidence interval were calculated for the standardized difference between the sarcopenic and healthy groups, with positive values indicating higher values in the sarcopenic group. All p values and effect sizes represent the reported between-group comparisons. Bold values indicate statistical significance at p<0.05 .

*Abbreviations:* 5STS, five-times sit-to-stand. CV, coefficient of variation.

**FIGURE 4:**
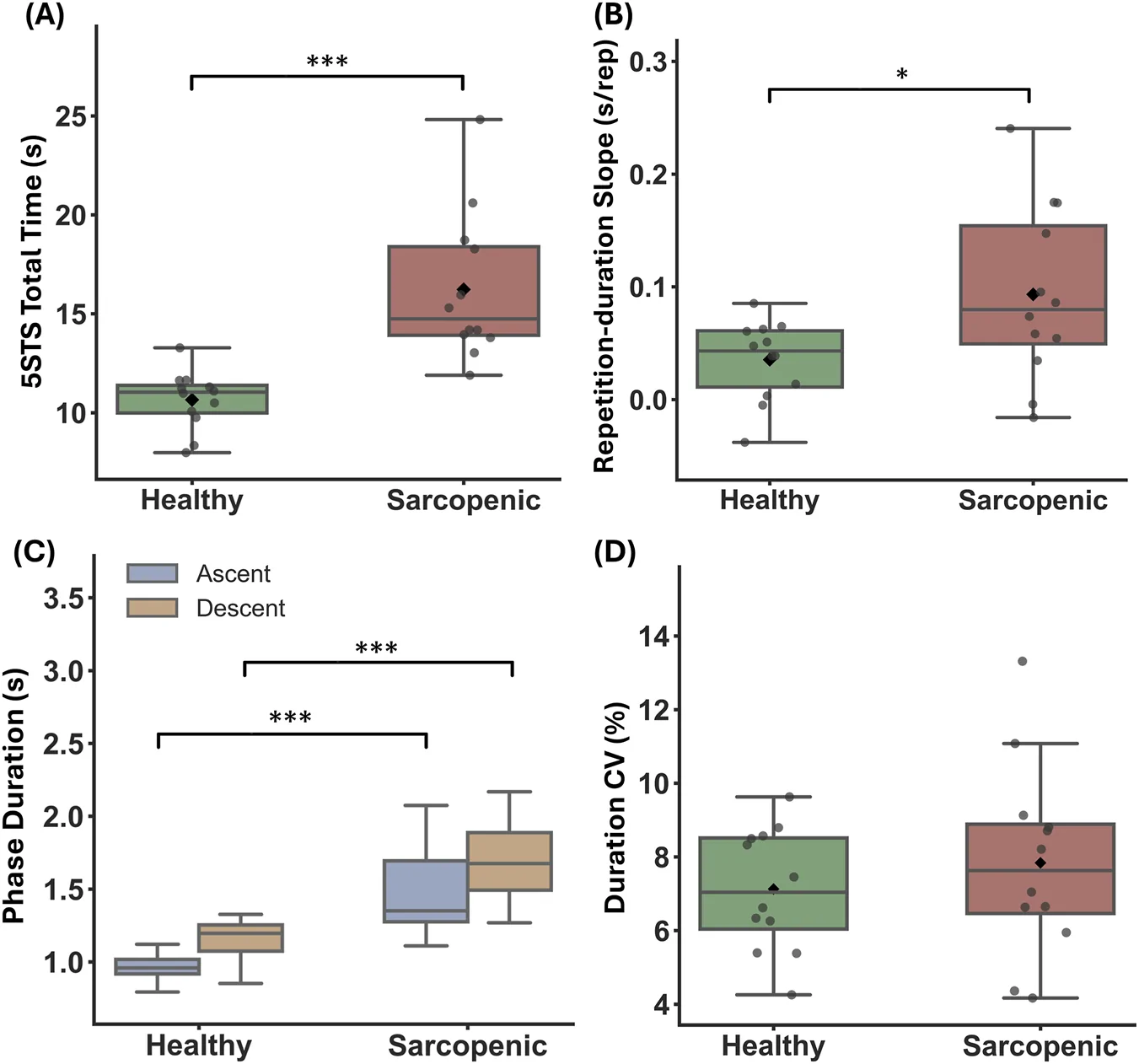
Between-group differences in OpenCap time-related 5STS performance metrics. Boxplots show **(A)** total 5STS completion time, **(B)** repetition-duration slope, **(C)** mean ascent and descent phase durations, and **(D)** repetition-duration coefficient of variation for the healthy and sarcopenic groups. In panels A, B, and D, the left and right boxes represent the healthy and sarcopenic groups, respectively, as indicated by the x-axis labels. In panel C, within each group, the left and right boxes represent ascent and descent durations, respectively. Thus, all categories can be identified by horizontal position and the x-axis group labels without relying on color. Boxes indicate the interquartile range. Horizontal lines indicate medians, whiskers represent 1.5 times the interquartile range, individual points represent participants, and black diamonds denote group means. Repetition-duration slope quantifies the linear change in repetition duration across the five repetitions, with larger positive values indicating a greater increase in duration across successive repetitions. Significance markers correspond to the between-group comparisons reported in Table 3; * indicates p<0.05 , and *∗∗ indicates p<0.001 .

Mean ascent duration was longer in the sarcopenic group than in the healthy group ( 1.48±0.33 s vs. 0.98±0.16 s, p<0.001 , Hedges’ g=1.89 , 95% CI 0.93–2.85). Mean descent duration was also longer in the sarcopenic group ( 1.76±0.44 s vs. 1.15±0.16 s, p<0.001 , Hedges’ g=1.77 , 95% CI 0.83–2.71). Repetition-duration CV did not differ significantly between the sarcopenic and healthy groups ( 7.84±2.64% vs. 7.13±1.66% , p=0.438 , Hedges’ g=0.31 , 95% CI −0.49 –1.12).

## 4 Discussion

The present study addressed two complementary questions during repeated 5STS in older Asian women. OpenCap showed strong participant-level agreement with Vicon for hip and knee flexion/extension, whereas ankle agreement was more variable. The known-groups analysis further identified differences in total and phase-specific timing, repetition-related slowing, and localized hip and knee kinematics between healthy and sarcopenic participants. The contribution of this study lies in the task- and population-specific validation of an established markerless motion capture platform and its use for joint-, phase-, and repetition-level characterization of 5STS performance. This extends previous OpenCap validation across gait and other functional tasks and complements video-based approaches to sarcopenia assessment (Uhlrich et al., 2023; Turner et al., 2024; Svetek et al., 2025; Ruiz-Cárdenas et al., 2023).

The validation results showed a joint-specific measurement profile. Knee flexion/extension had the lowest absolute error and the strongest agreement, while hip flexion/extension showed high waveform agreement with larger absolute errors. Ankle errors were relatively small in magnitude, but relative agreement was lower and more variable. This pattern reinforces the need to interpret RMSE, MAE, ICC(A,1), $R^{2}$ , mean bias, limits of agreement, and phase-dependent bias together. High ICC values can occur despite non-negligible angular error because ICC depends partly on waveform variability. Similarly, the lower ankle ICC may partly reflect the limited ankle excursion during 5STS. Previous OpenCap studies have also reported task- and joint-specific measurement performance (Uhlrich et al., 2023; Horsak et al., 2023; Turner et al., 2024; Svetek et al., 2025; Wang et al., 2025). The present results therefore apply to this repeated 5STS protocol and capture configuration.

Two features of the validation procedure also affect interpretation. First, OpenCap and Vicon were temporally aligned using trial-specific cross-correlation of the knee waveform because the systems did not share a hardware synchronization signal. The alignment corrected a rigid temporal offset and did not alter waveform amplitude or temporal scaling. However, use of the knee waveform for both alignment and subsequent validation could favor knee waveform agreement. Sensitivity to an independently synchronized or event-based alignment method was not evaluated. Second, agreement was calculated from participant-level mean waveforms after averaging valid repetitions and trials. This approach reduces within-participant random variability and is appropriate for the participant-level analysis used here, but it can produce more favorable agreement than single-repetition or single-trial comparisons. The reported agreement should be interpreted at the participant-mean waveform level.

The temporal analysis provided a more detailed description of how 5STS performance differed between groups. Total completion time showed clear group separation, while phase-specific analysis identified slowing during both rising and controlled lowering. These measures describe where time is spent within the task and can distinguish different components of performance that are combined in a single stopwatch measure. Individuals with similar total completion times could differ in rising duration, lowering duration, progressive slowing, or joint-movement strategy. The present study did not test whether these additional measures improve diagnosis, prognosis, or treatment selection beyond conventional 5STS timing. In addition, ascent duration, descent duration, repetition-duration slope, and repetition-duration variability were derived from OpenCap and were not independently validated against an external temporal reference. Their incremental clinical value and criterion validity therefore require further evaluation.

The prolonged ascent and descent durations provide complementary functional information. Rising requires lower-limb extensor force generation, body-mass transfer, and postural stabilization (Schenkman et al., 1990; Sadeh et al., 2023). Slower ascent is compatible with reduced lower-limb functional reserve or reduced capacity to generate force and power rapidly. These mechanisms were not measured directly in this study. Controlled descent places greater emphasis on eccentric control, trunk regulation, and postural stability (Jeon et al., 2021). A slower descent could reflect reduced eccentric capacity, greater postural demand, or a more cautious movement strategy. Fear of falling could also influence lowering behavior. Eccentric strength, postural strategy, and fear of falling were not directly assessed, so these explanations remain hypotheses.

The repetition-duration results add a different dimension to the phase-specific findings. The sarcopenic group showed greater progressive slowing across successive repetitions, while repetition-duration variability was similar between groups. This combination is more consistent with a systematic change in pace than with greater temporal irregularity. Reduced functional reserve, fatigability, lower local muscular endurance, and increasing neuromuscular demand are plausible explanations (Smith et al., 2020). The current data cannot distinguish among these mechanisms. Future studies combining kinematics with EMG could determine whether progressive slowing is accompanied by changes in muscle recruitment. More broadly, multimodal sensing that combines movement and physiological information has been explored for quantitative musculoskeletal monitoring during rehabilitation (Amin et al., 2025). Such measurements could complement OpenCap when the research question requires direct assessment of neuromuscular mechanisms.

The waveform analysis localized group differences to specific regions of the sit–stand–sit cycle. Hip differences extended across broader early and late regions, whereas the knee differences were confined to very brief intervals. The knee findings should be interpreted cautiously and require replication in a larger sample. Lower hip flexion near movement initiation and termination could reflect differences in trunk-momentum transfer or postural strategy, although trunk and pelvic kinematics were not analyzed directly. Hip and knee angular velocity waveforms did not differ significantly between groups. The longer movement durations observed in the sarcopenic group could reduce instantaneous velocity peaks by distributing angular displacement over a longer period, but this remains a possible explanation. Overall, the temporal outcomes provided clearer group separation in this sample than the localized waveform differences. Future work could extend the analysis to trunk and pelvic motion, interlimb asymmetry, and movement outside the sagittal plane.

The participant characteristics also define the scope of these findings. Both groups were relatively lean, and BMI and body weight did not differ significantly despite differences in ASM, SMI, handgrip strength, and calf circumference. The healthy group also had an average SMI close to the AWGS female cut-off. These characteristics indicate that the present comparison involved a relatively lean cohort within a narrow anthropometric range. The findings should therefore be generalized cautiously to men, other ethnic groups, individuals with higher adiposity, and people with sarcopenic obesity. Sarcopenia and frailty should also be distinguished conceptually. The present study classified participants according to AWGS sarcopenia criteria and did not conduct a standardized frailty assessment. A more comprehensive assessment of 5STS function could also consider the physiological resources required to perform and recover from the task, together with the participant’s subjective response. Individuals with similar movement performance may differ in physiological cost, perceived exertion, fatigue, or pain, which could indicate differences in functional reserve that are not captured by movement performance alone. Future studies integrating performance, physiological cost, and perception may therefore provide a more complete basis for functional assessment and intervention planning.

The present findings support further evaluation of OpenCap as a biomechanical measurement approach for repeated 5STS. They do not establish screening accuracy, diagnostic utility, or implementation feasibility in community or primary-care settings. These applications require evidence on repeatability, clinically meaningful thresholds, responsiveness, processing time, operator requirements, acceptability, and technical reliability. The exclusion of eight trials because of OpenCap synchronization-related pose-tracking failure also highlights the importance of evaluating technical failure rates under real-world conditions. Prospective implementation studies are needed before routine clinical use can be considered.

Several limitations should be considered. The sample was small and included only older Asian women. The between-group analyses were unadjusted, and the sample size did not support multivariable control for physical activity, medication use, pain, balance problems, or other potential confounders. The observed differences should be interpreted as group-associated patterns within this sample. The cross-sectional design does not support causal inference. Lower-limb strength, joint kinetics, muscle activation, muscle quality, and physiological cost were not directly measured, which limits mechanistic interpretation. The OpenCap-derived temporal measures were not independently validated against an external temporal reference. Participant-level waveform averaging reduced within-participant variability, so the agreement estimates should not be extrapolated to individual repetitions or trials. Finally, temporal alignment relied on cross-correlation of the knee waveform without hardware synchronization. An independent synchronization method and sensitivity analysis would strengthen future validation studies.

In conclusion, within this repeated 5STS protocol in older Asian women, OpenCap showed strong participant-level agreement with Vicon for hip and knee flexion/extension kinematics, while ankle agreement was more variable. OpenCap-derived analyses also identified phase-specific temporal, repetition-related, and localized kinematic differences between healthy and sarcopenic groups. These findings support task-specific biomechanical characterization of 5STS performance, while further studies are needed to establish the independent validity of derived temporal measures, repeatability, clinical utility, feasibility, and generalizability to broader populations.

## Data Availability

The raw data supporting the conclusions of this article will be made available by the authors, without undue reservation.
