# Supplementary material for: OpenCap-based five-times sit-to-stand analysis in older Asian women with and without sarcopenia
Source: Front Aging. 2026 Oct 5;7:1960201. doi: 10.3389/fragi.2026.1960201 (PMC13691588; doi:10.3389/fragi.2026.1960201)
Supplement: Supplementary file 1 [file DataSheet1.pdf]

Supplementary Data S1

1 SCOPE AND ANALYSIS LEVEL

This file provides participant-level OpenCap–Vicon agreement results referred to in the main manuscript but not presented in the main tables. It includes numerical mean bias and limits of agreement and the complete distribution of participant-level ICC(A,1) values. The healthy and sarcopenic groups each contained 12 participants.

All valid time-normalized repetitions were averaged within each participant before agreement metrics were calculated. Mean bias was defined as OpenCap minus Vicon. Limits of agreement (LoA) were calculated from the distribution of participant-level mean biases as the group mean bias ± 1.96 SD. Confidence intervals were obtained by bias-corrected and accelerated (BCa) bootstrap resampling with 10,000 resamples and participants as the resampling unit.

2 PARTICIPANT-LEVEL OPENCAP–VICON AGREEMENT

Table S1. Participant-level mean bias and numerical limits of agreement.

Group	Joint	Mean bias, °	Lower LoA, °	Upper LoA, °
Healthy	Hip	0.16 (0.09 to 0.22)	-0.07 (-0.15 to 0.02)	0.38 (0.30 to 0.46)
Healthy	Knee	-0.01 (-0.03 to 0.01)	-0.09 (-0.12 to -0.06)	0.06 (0.04 to 0.10)
Healthy	Ankle	-0.05 (-0.06 to -0.04)	-0.09 (-0.10 to -0.07)	-0.01 (-0.02 to 0.01)
Sarcopenic	Hip	0.22 (0.17 to 0.29)	0.01 (-0.06 to 0.07)	0.43 (0.31 to 0.56)
Sarcopenic	Knee	-0.03 (-0.04 to -0.01)	-0.09 (-0.11 to -0.06)	0.04 (0.02 to 0.06)
Sarcopenic	Ankle	-0.05 (-0.06 to -0.04)	-0.10 (-0.12 to -0.08)	0.00 (-0.02 to 0.02)
Overall	Hip	0.19 (0.14 to 0.23)	-0.03 (-0.11 to 0.02)	0.41 (0.34 to 0.51)
Overall	Knee	-0.02 (-0.03 to -0.01)	-0.09 (-0.11 to -0.07)	0.05 (0.03 to 0.07)
Overall	Ankle	-0.05 (-0.06 to -0.04)	-0.09 (-0.11 to -0.08)	0.00 (-0.02 to 0.01)

Note: Values are estimates with 95% CIs in parentheses. LoA were calculated across participant-level mean biases, rather than across the 101 phase points within one waveform. Positive values indicate that OpenCap produced a larger angle than Vicon. Abbreviation: LoA, limits of agreement.

Table S2: Complete participant-level distribution of ICC(A,1) values.

Participant	Group	Hip ICC(A,1)	Knee ICC(A,1)	Ankle ICC(A,1)
S21	Healthy	0.973 (excellent)	0.999 (excellent)	0.883 (good)
S22	Sarcopenic	0.972 (excellent)	0.995 (excellent)	0.124 (poor)
S23	Sarcopenic	0.981 (excellent)	0.997 (excellent)	0.858 (good)
S24	Sarcopenic	0.975 (excellent)	0.993 (excellent)	0.732 (moderate)
S25	Sarcopenic	0.990 (excellent)	0.997 (excellent)	0.812 (good)
S26	Healthy	0.985 (excellent)	0.999 (excellent)	0.942 (excellent)
S27	Sarcopenic	0.923 (excellent)	0.999 (excellent)	0.909 (excellent)
S28	Sarcopenic	0.958 (excellent)	0.994 (excellent)	0.543 (moderate)
S29	Healthy	0.982 (excellent)	1.000 (excellent)	0.984 (excellent)
S30	Sarcopenic	0.896 (good)	0.998 (excellent)	0.980 (excellent)

Continued on next page

Table S2 continued

Participant	Group	Hip ICC(A,1)	Knee ICC(A,1)	Ankle ICC(A,1)
S31	Healthy	0.974 (excellent)	0.998 (excellent)	0.986 (excellent)
S32	Healthy	0.978 (excellent)	0.998 (excellent)	0.955 (excellent)
S33	Healthy	0.981 (excellent)	0.994 (excellent)	0.923 (excellent)
S34	Healthy	0.980 (excellent)	0.999 (excellent)	0.983 (excellent)
S35	Healthy	0.937 (excellent)	1.000 (excellent)	0.992 (excellent)
S36	Healthy	0.968 (excellent)	0.998 (excellent)	0.977 (excellent)
S37	Sarcopenic	0.986 (excellent)	0.998 (excellent)	0.984 (excellent)
S38	Sarcopenic	0.980 (excellent)	0.996 (excellent)	0.814 (good)
S39	Sarcopenic	0.946 (excellent)	0.999 (excellent)	0.983 (excellent)
S40	Sarcopenic	0.964 (excellent)	0.998 (excellent)	0.980 (excellent)
S41	Healthy	0.980 (excellent)	0.998 (excellent)	0.872 (good)
S42	Sarcopenic	0.975 (excellent)	0.999 (excellent)	0.990 (excellent)
S43	Healthy	0.996 (excellent)	0.996 (excellent)	0.961 (excellent)
S44	Healthy	0.991 (excellent)	0.998 (excellent)	0.976 (excellent)

Note: Participant codes are pseudonymous. Interpretive categories were poor (< 0.50), moderate (0.50–0.74), good (0.75–0.90), and excellent (> 0.90). In the healthy group, the ranges were 0.937–0.996 for the hip, 0.994–1.000 for the knee, and 0.872–0.992 for the ankle. In the sarcopenic group, the corresponding ranges were 0.896–0.990, 0.993–0.999, and 0.124–0.990.
